# Co-Occurrence of Hypoglycin A and Hypoglycin B in Sycamore and Box Elder Maple Proved by LC-MS/MS and LC-HR-MS

**DOI:** 10.3390/toxins14090608

**Published:** 2022-09-01

**Authors:** Ahmed H. El-Khatib, Anna Maria Engel, Stefan Weigel

**Affiliations:** German Federal Institute for Risk Assessment (BfR), Department for Safety in the Food Chain, Max-Dohrn-Str. 8-10, 10589 Berlin, Germany

**Keywords:** *Acer pseudoplatanus*, *Acer negundo*, atypical myopathy, HRMS, structure elucidation, mzLogic, in silico fragmentation

## Abstract

Hypoglycin A (HGA) and methylenecyclpropylglycine (MCPrG) are formed by some maple trees (*Acer* species) and have been associated with incidences of atypical myopathy among horses in pastures. In this work, a simple and sensitive ultra-performance liquid chromatography tandem mass spectrometry (UPLC–MS/MS) method without derivatization was developed for the quantification of HGA and MCPrG in maple samples and validated according to EU guidelines. The LOQ presented here for HGA (16.4 µg/kg) is considerably lower than the lowest published LOQ (500 µg/kg). This method confirms that sycamore and box elder maple contain considerable amounts of HGA and MCPrG. In addition, the presence of the dipeptides hypoglycin B and γ-glutamyl-MCPrG in these two maple species is shown using high-resolution MS. This is the first report on the presence of these dipeptides in maple since 1973. The presence of HGB and γ-glutamyl-MCPrG could change the way we understand animal intoxication following the ingestion of maple.

## 1. Introduction

Hypoglycin A (HGA, methylenecyclpropylalanine) and its homologue methylenecyclpropylglycine (MCPrG) are naturally occurring non-proteinogenic toxic amino acids [1]. They are known to exist in high concentrations in some plants of the family Sapindaceae such as unripe lychee and ackee fruits [2,3,4,5,6] and in the seeds, leaves and seedlings/young shoots of some maple trees (*Acer* species) such as sycamore maple (*Acer pseudoplatanus*) [7,8,9,10] and box elder maple (*Acer negundo*) [11]. Being toxic to many species, HGA and MCPrG were identified to be associated with diseases such as hypoglycemic encephalopathy [12,13] and Jamaican vomiting sickness [14,15,16,17,18] in humans and atypical myopathy (AM) in horses [19,20,21,22,23], deer [24,25] and Bactrian camels [26]. Due to the abundance of maple in central Europe and the United States, among other places, there is a risk of animal intoxication following the ingestion of maple seeds and seedlings. These toxins have been proven to pass into mares’ milk [27,28]. In addition, there are indications that transfer into the milk of cows may occur [29] possibly posing a health risk if contaminated milk is ingested by humans.

HGA and MCPrG are not toxic per se, but they are bioactivated in humans through the metabolism into the coenzyme A (CoA) adducts of methylenecyclopropylacetic acid (MCPA) and methylenecyclopropylformic acid (MCPF), respectively. MCPA-CoA and MCPF-CoA bind to the multiple acyl-CoA dehydrogenases that are necessary for the metabolism of short- and medium-chain fatty acids and branched-chain amino acids. This leads to the inability to metabolize fatty acids and the accumulation of fat esters that damage the muscle cell membrane, triggering the symptoms of intoxication [23,30,31]. In addition, the reduction in fatty acid metabolism causes an increased use of glucose and the blockade of the substrate for hepatic gluconeogenesis, leading eventually to hypoglycemia after the hepatic glycogen stores are depleted [32,33].

**Figure 1:**
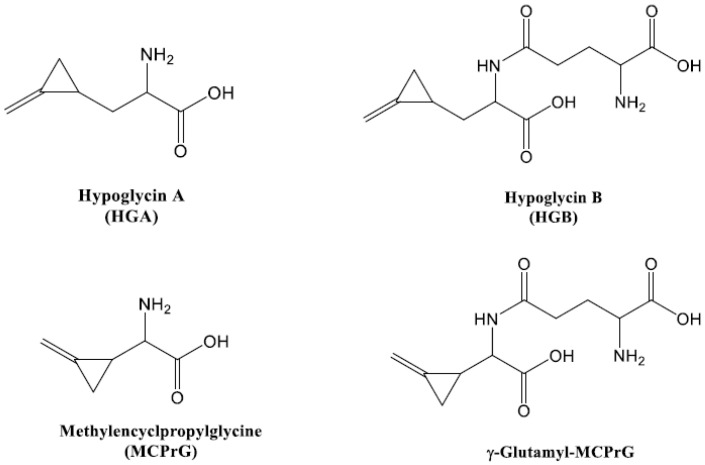
Chemical structures of the toxins investigated in this study.

Other toxins such as hypoglycin B (γ-glutamyl-hypoglycin, HGB) and γ-glutamyl-α-(methylenecyclopropyl)glycine (γ-glutamyl-MCPrG) have been also reported to exist in the seeds of ackee fruits. They are the dipeptides of glutamic acid and HGA and MCPrG, respectively. HGB and γ-glutamyl-MCPrG show lower hypoglycemic activity than HGA and MCPrG but are still associated with Jamaican vomiting sickness [3,34,35]. Few reports and reviews have been published on the occurrence of HGB and γ-glutamyl-MCPrG in the seeds of *Acer pseudoplatanus* [36,37]. The structures of HGA, MCPrG, HGB and γ-glutamyl-MCPrG are shown in Figure 1.

Several LC-MS methods for the quantification of HGA and MCPrG in different plant materials and HGB in ackee have been reported. The majority of these methods involve pre-column derivatization with o-phthalaldehyde [38,39], phenylisothiocyanate [3,40], butylation (3N HCl in n-Butanol) [41] or dansyl chloride [4]. Fewer methods have demonstrated the LC-MS quantification of HGA and MCPrG without derivatization [6,9,10]. In the latest study performed on maple species, Medina et al. have reported a limit of quantification (LOQ) of 500 µg/kg for HGA [10].

In this work, a simple, sensitive and validated ultra-performance liquid chromatography tandem mass spectrometry (UPLC–MS/MS) method without derivatization for the quantification of HGA and MCPrG in *Acer* species was developed and applied for the screening of maple leaves, seeds and seedlings for the presence and levels of these compounds. In addition, the presence of the dipeptides HGB and γ-glutamyl-MCPrG in two *Acer* species was confirmed using high-resolution (HR) MS/MS. The confirmation methodology included matching HR fragmentation data with available standards/databases and systematic structure elucidation using the mzLogic algorithm and in silico fragmentation. This is the first report on the presence of HGB and γ-glutamyl-MCPrG in maple since the work of Fowden and Pratt in 1973 [36].

## 2. Results and Discussion

### 2.1. Sample Selection

Seeds, leaves and seedlings from different *Acer* species (*A. pseudoplatanus* (sycamore maple), *A. negundo* (box elder maple), *A. campestre* (field maple), *A. platanoides* (Norway maple), *A. tataricum* (Tatar maple) and *A. cappadocicum* (Cappadocian maple)) were screened for the presence of HGA and MCPrG using HRMS. HGA and MCPrG were not detected in *A. campestre*, *A. platanoides*, *A. tataricum* and *A. cappadocicum*. On the other hand, *A. pseudoplatanus* and *A. negundo* showed distinct signals for the toxins. Therefore, method development and validation were performed using the seeds of *A. platanoides* as a blank matrix. The validated method was finally applied for the quantification of HGA and MCPrG in *A. pseudoplatanus* and *A. negundo* samples.

### 2.2. Extraction Optimization

The number of extraction cycles (up to three cycles) was investigated using three solvents (water, 1% formic acid in water and methanol) on the naturally contaminated *A. pseudoplatanus* seeds. The extraction efficiency using water and acidified water was noticeably better than using methanol. The extraction with water was preferred due to the higher extraction efficiency. The first two extraction fractions proved to contain more than 99% of the extracted HGA (Figure S1). Therefore, only two extraction cycles with water were implemented in the presented method.

### 2.3. Method Performance and Validation

**Figure 2:**
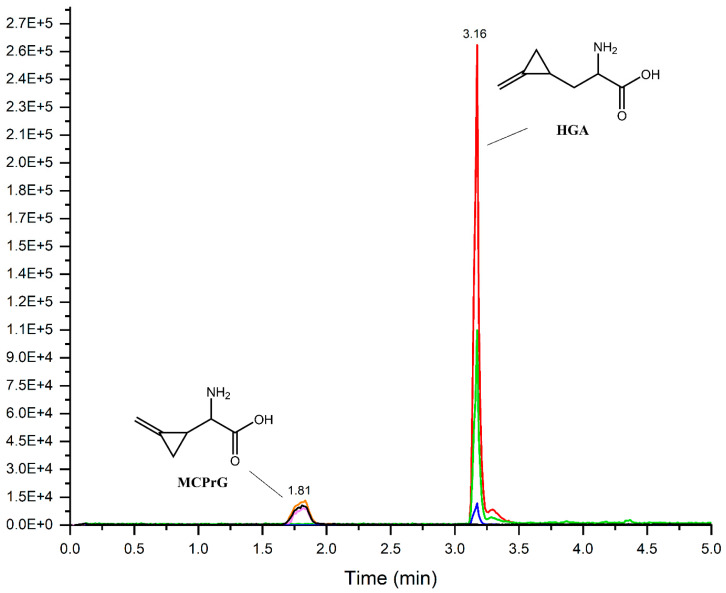
Overlaid MRM-extracted ion chromatograms of HGA and MCPrG in spiked Norway maple (*A. platanoides*) seeds sample.

In this work, a simple LC-MS/MS method without derivatization was developed, optimized and validated for the quantification of HGA and MCPrG in maple samples. The MRM-extracted ion chromatograms of HGA and MCPrG are shown in Figure 2. The method is linear in the range of 0.5–100 ng/mL (equivalent to 10–2000 µg toxin/kg plant material) for both HGA and MCPrG. The LOD was 5.0 and 6.4 µg/kg for HGA and MCPrG, respectively. The LOQ was 16.4 µg/kg for HGA and 21.2 µg/kg for MCPrG. The LOQ presented here (16.4 µg/kg) is noticeably lower than the LOQ reported by Medina et al. (500 µg/kg) for HGA [10]. Good values for recovery (90–105% for HGA and 94–105% for MCPrG) and precision (≤20%) were obtained for all the QC levels. The lowest validated level according to SANTE/2019/12682 guidelines is therefore 20 µg/kg for both HGA and MCPrG. The validation results were generally meeting validation requirements according to the SANTE guidelines and are summarized in Table 1.

The matrix effect was 48% and 53% for HGA and MCPrG, respectively. No matrix effect was observed when the extract was 1 to 25 diluted with 5% MeOH/water before LC-MS/MS measurement (Figure S2). The dilution approach showed also good validation results (Table S2). This 25-fold dilution will, however, raise the lowest validated level of the method to 500 µg/kg. Therefore, the dilution approach would be only suitable for samples that contain sufficiently high levels of HGA and MCPrG.

### 2.4. HGA and MCPrG in Field Samples

Of the collected samples from different *Acer* species in Germany, only the samples of sycamore maple (*A. pseudoplatanus*) and box elder maple (*A. negundo*) showed measurable quantities of HGA and MCPrG (Table 2). The data show that the investigated sycamore maple seedlings contain HGA (range 3223–4508 mg/kg) and MCPrG (range 290–500 mg/kg), while the seeds contained 266–2962 and 35–267 mg/kg for HGA and MCPrG, respectively. In the investigated sycamore leaves, the ranges were 120–3202 and 6.3–158 mg/kg for HGA and MCPrG, respectively. Similarly, the investigated box elder maple seeds contain HGA and MCPrG in the range of 236–584 and 22–56 mg/kg, respectively, while the leaves contain 24–1047 and 2.7–4.1 mg/kg for HGA and MCPrG, respectively. Although these findings could be limited by the small sample size (n = 2) for sycamore seedlings and box elder samples, these results are in agreement with other studies conducted on sycamore maple samples in other European countries (2–9120, 5–271 and 97–3400 mg HGA/kg seeds, leaves and seedlings, respectively) [7,8,9,10] and box elder maple in the USA (10–510 mg HGA/kg seeds) [11]. The variable concentrations of HGA and MCPrG in different species, in different plant parts among trees (Table 2) and within the same tree (Table S3) could be attributed to several factors, such as the maturity of seeds, soil composition, temperature fluctuation, stress, sunlight and rainfall, among others [3,11]. As an example, the unripe seeds of one sycamore tree contain less HGA (303 mg/kg) than the leaves contain (459 mg/kg) taken from the same tree (sample #4, Table S3). These findings confirm that the trees of *A. pseudoplatanus* (native to Europe) and *A. negundo* (native to North America) are the only source of HGA among the investigated *Acer* species. The ingestion of the seeds and seedlings of *A. pseudoplatanus* is therefore associated with incidences of AM among horses on European pastures [22,42]. The determination of HGA, MCPrG and their metabolites in the blood and urine of horses is a useful tool for screening for exposure to these toxins and probably also for prophylaxis against AM before the manifestation of clinical symptoms [8,21]. Grazing on sycamore maple seedlings has resulted in serum HGA levels in sheep similar to those in the serum of horses with subclinical AM [43], while there were no clinical signs of poisoning in sheep. Although the intoxication of wild ruminants has been demonstrated [24,25], it is not known whether serum concentrations similar to those of intoxicated horses can cause clinical signs in domestic ruminants. In addition, HGA excretion in milk is possible to occur, as recently demonstrated by the detection of HGA in nursing lambs [43] and cow’s milk [29]. In order to evaluate possible health risks to humans, reliable data on the presence and levels of HGA in milk and dairy products would be needed.

### 2.5. Detection of HGB and γ-Glutamyl-MCPrG in Sycamore and Box Elder Maple Samples

**Figure 3:**
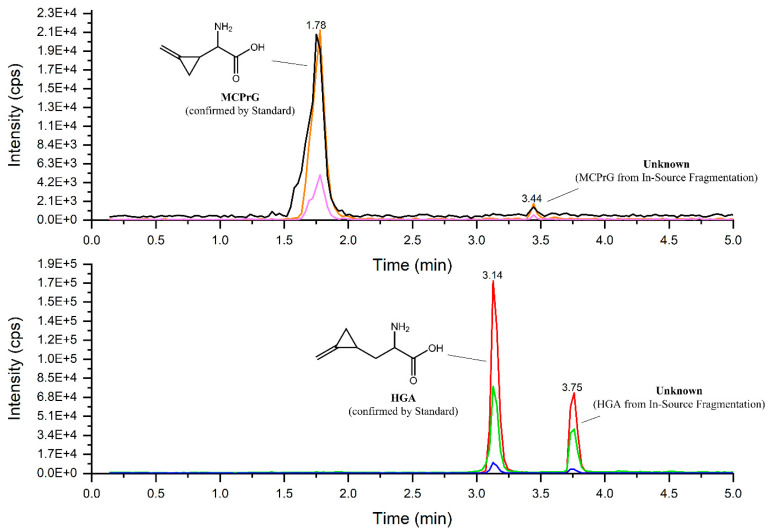
Overlaid MRM-extracted ion chromatograms of HGA (**lower** panel) and MCPrG (**upper** panel) in sycamore maple (*A. pseudoplatanus*) seeds.

**Figure 4:**
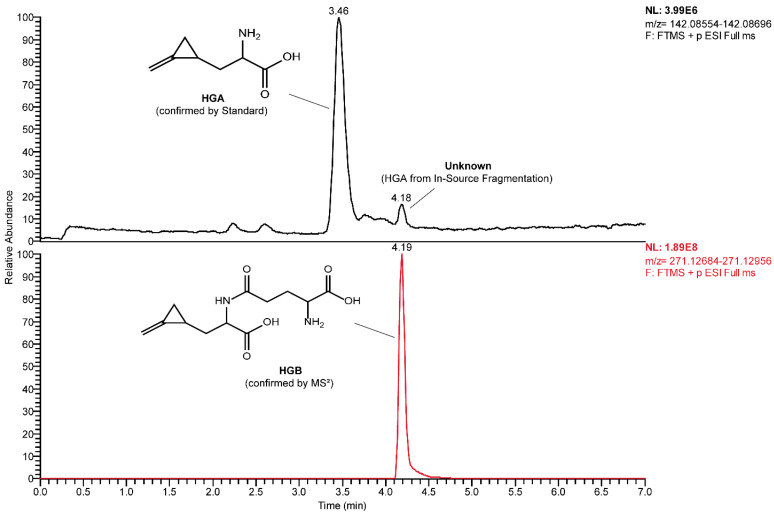
HRMS-extracted ion chromatograms of HGA (**upper** panel) and HGB (**lower** panel) in sycamore maple (*A. pseudoplatanus*) seeds.

**Figure 5:**
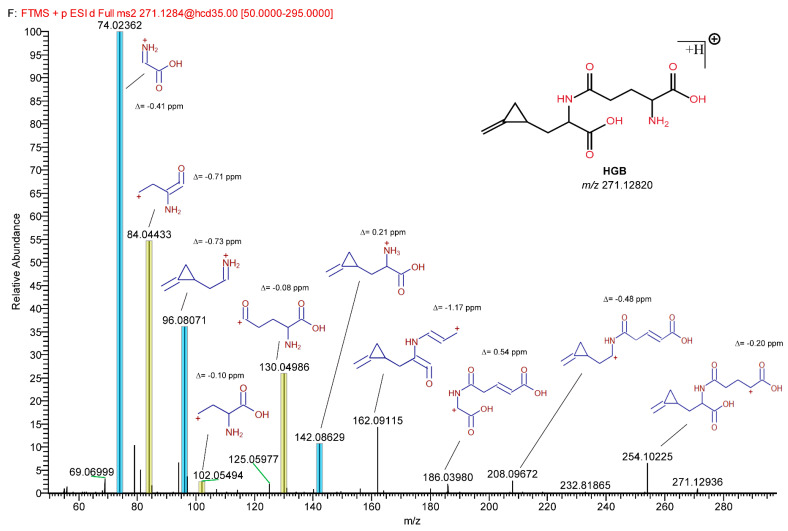
Structure elucidation using in silico fragmentation and fragment ion search (FISh) analysis of HGB. Fragments in the query HRMS/MS spectrum were explained and structurally annotated using general fragmentation rules. The mass accuracy (∆ ppm) is shown for each fragment. The blue- and yellow-highlighted fragments are characteristic of HGA and glutamic acid, respectively.

The MRM-extracted ion chromatogram of HGA in sycamore maple samples showed another peak at a higher retention time showing all three characteristic MRM transitions of HGA and MCPrG in sycamore maple seeds (Figure 3) and seedlings (Figure S3). The explanation of this could be the in-source fragmentation of a compound that contains HGA or MCPrG, respectively. The samples were re-analyzed using HRMS in positive ionization mode and the *m*/*z* values for those peaks were identified (271.12820 and 257.11264 for compounds probably containing HGA (Figure 4) and MCPrG (Figure S4), respectively). Within a mass tolerance of 3 ppm, these *m*/*z* values were matching those of HGB and γ-glutamyl-MCPrG, respectively. The MS^2^ spectra showed fragments that are characteristic for HGA, MCPrG and glutamic acid, as confirmed against the MS^2^ spectra of standard HGA, MCPrG and glutamic acid and/or the mzCloud HRMS spectral database (the mzCloud database does not include reference MS^2^ spectra for HGB or γ-glutamyl-MCPrG). In addition, systematic structure elucidation using HRMS/MS data was conducted using the mzLogic algorithm, which utilizes elemental composition to search structural databases in addition to extensive high-resolution fragmentation information from mzCloud and then ranks potential structural candidates according to spectral similarity and sub-structural information. Running mzLogic resulted in HGB as the top ranked candidate (Figure S5). Furthermore, the in silico fragmentation of HGB and γ-glutamyl-MCPrG structures using general fragmentation rules and fragment ion search (FISh) analysis explained and structurally annotated all fragments in the respective MS^2^ spectra with an excellent mass accuracy (Figure 5 and Figure S6 for HGB and γ-glutamyl-MCPrG, respectively). Similar chromatograms were observed in all seed, leaf and seedling samples of sycamore and box elder maple. This corroborates that different plant parts of sycamore and box elder maple contain the dipeptides HGB and γ-glutamyl-MCPrG. These dipeptides were not detected in Norway maple samples. In the pioneering work of Fowden and Pratt, the presence of HGB and γ-glutamyl-MCPrG in sycamore and box elder maple, among others, was confirmed using infrared, paper chromatography and electrophoretic techniques [36]. The study at hand is the first study to confirm the presence of these dipeptides in maple using high-performance liquid chromatography and mass spectrometry (LC-HRMS). Due to the unavailability of reference HGB and γ-glutamyl-MCPrG standards, the unequivocal confirmation and quantification of HGB and γ-glutamyl-MCPrG was not possible. Due to the possible differences in the electrospray ionization efficiency of HGA and MCPrG and their respective dipeptides, a direct estimation of the concentrations of the dipeptides based on their peak areas was not possible. However, the relative peak areas could still be used to explore the data. The comparison of peak areas showed that the investigated sycamore and box elder seeds and seedlings contained relatively more HGB and γ-glutamyl-MCPrG than leaves did (Table 2). In addition, the HGB/HGA peak area ratio showed a relatively low value in sycamore leaves (range 0.08–1.1) compared to seeds (0.03–29), indicating a possible higher extent of the formation of HGB in the seeds. This pattern was also observed in leaves and seeds samples of the same sycamore maple trees (Table S3). In the sycamore seedlings, the HGB/HGA ratio was 1.2–1.3. Similar findings could be also observed in the box elder seeds and leaves (Table 2 and Table S3). The samples of the same trees also showed higher γ-glutamyl-MCPrG/MCPrG peak area ratios in seeds than in leaves of sycamore (three out of four trees) and box elder maple (Table S3). It is worth mentioning that, in the samples from that single sycamore tree showing a lower γ-glutamyl-MCPrG/MCPrG peak area ratio in seeds than in leaves, the seeds were not ripe.

HGB and γ-glutamyl-MCPrG are known to be present in the seeds of ripe ackee fruit. It was demonstrated that an inverse relationship exists between the levels of HGA and HGB in the seeds during the ripening process of ackee fruit. During ripening, the amount of HGA decreases while HGB level increases. This formation of HGB from HGA is believed to be mediated by the enzyme γ-glutamyl-transpeptidase (also known as γ-glutamyl-transferase) in the biosynthetic pathway [44]. HGB is considered as a reservoir for HGA [3]. The enzyme γ-glutamyl-transpeptidase was also detected in *Acer pseudoplatanus* (a member of the family Sapindaceae like ackee) [45].

To the best of our knowledge, this is the first report on the presence of HGB and γ-glutamyl-MCPrG in maple in the past 50 years. In 1973, Fowden and Pratt detected HGB and γ-glutamyl-MCPrG in sycamore seeds [36]. Since then, the more toxic HGA has been the focus of studies, whereas HGB has been overlooked. Very little was found in the literature on the question of the effects of HGB on animal health. Judged from the lethal dose, HGB is less potent than HGA in rabbits, mice and rats [46]. In the conducted studies, 25 mg HGB/kg bodyweight caused death after the intravenous injection of HGB in rabbits, which represented the most sensitive animals in the study. Histopathological examinations led to the discovery of the fatty metamorphosis of the kidney, pulmonary edema as well as the erosions of the gastric mucosa. In addition, HGB was proven to show hypoglycemic actions. A previous study in rats reported that following intrauterine injection of 100 µg HGB mixed with 0.2 mL water, there was an increased incidence (88 out of 146 rats) of congenital malformations [47].

So far, the involvement of HGB in the development of atypical myopathy in horses has not been considered. This may be due to a lack of information on the presence and concentrations of HGB in seeds and seedlings as well as serum and/or urine samples. Further investigations are needed to understand the role of HGB in the process of poisoning and to draw possible conclusions to metabolic involvement. It is still unclear whether or to which extent HGB might transform in the gastro-intestinal tracts (GITs) or livers of farm animals. Upon ingestion by grazing animals, the hydrolysis of HGB into HGA cannot be excluded. The presence of HGB and γ-glutamyl-MCPrG could thus change the way we understand animal intoxication following the ingestion of maple. Therefore, more attention should be paid to HGB and γ-glutamyl-MCPrG, whose quantification should be integrated in the current methods of the analysis of HGA and MCPrG.

In summary, a simple, sensitive and validated method for the quantification of HGA and MCPrG in *Acer* species was developed and applied for the screening of maple leaves, seeds and seedlings for the presence and levels of these compounds. The method included a simple extraction procedure and UPLC-MS/MS analysis without derivatization. The method was validated according to the EU guidelines and demonstrated good recovery and precision for diluted and undiluted samples. The dilution approach has the advantage of eliminating the matrix effect. The sensitivity of the developed method (an LOQ of 16.4 µg/kg for HGA) proved to be better than for already published methods (500 µg/kg for HGA). In addition to the quantification of HGA and MCPrG in sycamore and box elder maple samples, the presence of the dipeptides HGB and γ-glutamyl-MCPrG in these two *Acer* species was confirmed using high-resolution (HR) MS/MS.

## 3. Conclusions

The method proposed in this work allows for the quick and sensitive quantification of HGA and MCPrG in maple samples. It can be also adapted to include the quantification of HGB and γ-glutamyl-MCPrG once reference standards are available. In addition to the detection of noticeable levels of HGA and MCPrG in sycamore and box elder maple samples, the HRMS/MS analyses confirm the presence of the dipeptides HGB and γ-glutamyl-MCPrG in maple at levels that are probably higher than those of HGA and MCPrG, respectively. These dipeptides have been overlooked in maple for decades. Further investigations are needed to examine the relevance and impact of the presence of HGB and γ-glutamyl-MCPrG on the course of intoxications as well as the potential risks to humans.

## 4. Materials and Methods

### 4.1. Chemicals and Standards

(S)-hypoglycin A (HGA, purity 85%) and α-(methylenecyclopropyl)glycine (MCPrG) standards were purchased from Toronto Research Chemicals (Toronto, ON, Canada). Acetonitrile (ACN), methanol (MeOH), formic acid (FA) and ammonium formate (NH_4_COOH) were purchased from Merck (Darmstadt, Germany). Double-deionized water was obtained using a Milli-Q system from Merck (Merck Millipore, Darmstadt, Germany).

### 4.2. Plant Materials

Seeds, leaves and seedlings/young shoots from different *Acer* species (*A. pseudoplatanus* (sycamore maple), *A. negundo* (box elder maple), *A. campestre* (field maple), *A. platanoides* (Norway maple), *A. tataricum* (Tatar maple) and *A. cappadocicum* (Cappadocian maple)) were collected from different sites in the Berlin-Brandenburg region in the north-east of Germany and North Rhine-Westphalia in the western part of Germany. The samples were air-dried and then homogenized using knife/ball mills (Retsch, Haan, Germany). The homogenized material was stored in a dry place at room temperature.

### 4.3. Sample Preparation

Five mL deionized water was added to 0.5 g homogenized plant material. The mixture was vortexed and then sonicated in an ultrasonic bath (Sonorex Super RK 100 H, Bendelin, Berlin, Germany) for 10 min (35 kHz, 80 W) at room temperature. Subsequently, the sample was centrifuged for 10 min at 4000 rpm (Heraeus Megafuge 16, Thermo Fisher Scientific, Waltham, MA, USA) at room temperature. The supernatant was then filtered (Ahlstrom Folded filters, NeoLab Heidelberg, Germany) into a new 15 mL Falcon tube. The residues of the extracted sample were extracted again with another 5 mL water as above, centrifuged, filtered and combined with the first extract supernatant. Samples (undiluted or 1 to 25 diluted with 5% methanol/water) were then measured by LC-MS/MS.

### 4.4. Stock and Working Standard Preparation

Stock HGA and MCPrG standard solutions (0.1 mg/mL) were prepared in 50% ACN in water (*v*/*v*). A working standard mixture (1.0 µg/mL) was prepared by mixing stock solutions and dilution with 5% MeOH in water (*v*/*v*). The mixture served both as a calibration mixture and as a spiking solution for validation. For calibration, a series of solutions at 0.5, 1, 2, 3, 4, 5, 10, 25, 50 and 100 ng/mL HGA and MCPrG were prepared in 5% MeOH and blank extract (matrix-matched calibration).

### 4.5. LC-MS/MS Instrumentation and Measurements

Analysis of extracted samples was performed on an Agilent 1290 Infinity II UPLC system, including binary pumps, a degasser, a column oven, an autosampler and a control unit (Agilent Technologies, Waldbronn, Germany), coupled to a Q-Trap 6500+ mass spectrometer (AB Sciex Germany GmbH, Darmstadt, Germany) equipped with an IonDrive™ Turbo V electrospray ionization (ESI) source. Chromatographic reversed-phase (RP) separation with 10 μL injection volume was achieved on a Waters Acquity UPLC BEH C18 column (150 × 2.1 mm, 1.7 μm particle size) with guard column (Waters, Milford, MA, USA) at a flow rate of 0.3 mL/min and a column oven temperature of 40 °C. The binary mobile phase consisted of 5 mM ammonium formate and 0.1% FA in water (eluent A) and methanol (eluent B). The gradient elution was adopted as follows: 0 min 0% B; 1 min 0% B; 2 min 50% B; 3 min 70% B; 5 min 100% B; 7 min 100% B; 7.5 min 0% B; 10 min 0% B. MS detection was conducted using positive electrospray ionization (ESI) and measuring in multiple reaction monitoring (MRM) mode with mass transitions and MS conditions shown in Table S1. The following instrumental settings were applied: curtain gas: 25; collision gas: medium; temperature: 500 °C; ion spray voltage: 5000 V; ion source gas 1:70; ion source gas 2:55. A diverter valve cut off the flow to the MS ion source before minute 0.1 and after minute 8.0. The MS/MS parameters used for quantification of HGA and MCPrG are listed in Table S1.

### 4.6. LC-HRMS/MS Instrumentation and Measurements

The samples were analyzed using an UltiMate 3000 UPLC coupled to a QExactive Focus mass spectrometer (Thermo Fisher, Dreieich, Germany) equipped with a heated electrospray ionization (HESI) source. Chromatographic reversed-phase (RP) separation was performed using the same conditions mentioned above. HRMS was performed in positive ionization mode. The HESI temperature was set at 400 °C, the capillary temperature at 256 °C, the electrospray voltage at 3.5 kV and S-Lens RF level at 60. Sheath and auxiliary gas flow rates were 47.5 and 11.25 L/min, respectively. All data in this study were acquired using a full scan mode covering the mass range from 80 to 500 *m*/*z* with a resolution of 70,000 and automatic gain control (AGC) setting of 3 × 10^6^ with a maximum injection time (IT) of 100 ms. For confirmation, data-dependent MS2 (dd-MS^2^) was applied. In dd-MS^2^, the most abundant precursor ions in each full scan were selected by the quadrupole and then sent to the higher-energy collisional dissociation (HCD) cell for ion fragmentation and finally to the Orbitrap mass analyzer for detection. The dd-MS^2^ was performed at a mass resolution of 17,500, intensity threshold of 6.0e^4^, isolation width of 1.0 *m*/*z* and normalized collision energy (NCE) of 35% with ±20% step. This method was used for the screening of different *Acer* species samples for the presence of HGA and MCPrG (full scan mode) and for the structure elucidation of HGB and γ-glutamyl-MCPrG (dd-MS^2^).

### 4.7. Method Validation

Samples from different *Acer* species were screened for the presence of HGA and MCPrG using HRMS. Seeds of *Acer platanoides* (Norway maple) were used as a blank matrix for method validation. The method was validated according to the European Union SANTE/2019/12682 guidelines [48]. The method validation parameters and performance criteria are as follows:

Linearity and range: a series of matrix-matched standard solutions in the range of 0.5–100 ng/mL HGA and MCPrG were evaluated. Deviation of back-calculated concentration from true concentration should be ≤±20%.

Limit of detection (LOD) and limit of quantification (LOQ): LOD and LOQ were estimated according to the EURL Guidance Document on the Estimation of LOD and LOQ for Measurements in the Field of Contaminants in Feed and Food [49] using spiked blank samples.

Recovery: four quality control (QC) samples were prepared by spiking and then extracting blank samples. The QC levels were: LOQ (20 µg/kg), low (QCL, 50 µg/kg), medium (QCM, 500 µg/kg) and high (QCH, 1500 µg/kg). The average recovery for each QC level should be within 70–120%.

Precision: repeatability (intra-day precision, RSD_r_) and within-laboratory reproducibility (inter-day precision, RSD_wR_) were estimated for the QC samples. RSD_r_ and RSD_wR_ for each QC level should be ≤20%.

Matrix effect: the response of the matrix-matched standard solutions was compared to that of standard solutions prepared in methanol.

### 4.8. Data Analysis

LC-MS/MS data evaluation was performed with MultiQuant Software, ver. 3.0.2 (AB Sciex Germany GmbH, Darmstadt, Germany). LC-HRMS/MS data evaluation and structure elucidation were performed with Xcalibur, ver. 4.4 and Mass Frontier, ver. 8.0 SR1 software (Thermo Fisher, Dreieich, Germany).

## Figures and Tables

**Table 1:** Method validation parameters for the determination of HGA and MCPrG in maple. LOD and LOQ are estimated using spiked blank material.

Parameter		HGA	MCPrG
**Calibration range** **(ng/mL)**		0.5–100	0.5–100
		(10–2000 µg/kg)	(10–2000 µg/kg)
**Correlation coefficient (r)**		0.9999	0.9999
**LOD (µg/kg)**		5.0	6.4
**LOQ (µg/kg)**		16.4	21.2
**Recovery (%)**	20 µg/kg	95.5	105.1
	50 µg/kg	104.7	99.6
	500 µg/kg	92.1	98.1
	1500 µg/kg	89.7	93.5
**Repeatability** **(RSD_r_) (%)**	20 µg/kg	6.5	7.8
	50 µg/kg	12.9	6.0
	500 µg/kg	2.6	4.3
	1500 µg/kg	13.2	1.0
**Within-laboratory reproducibility (RSD_wR_) (%)**	20 µg/kg	10.8	10.7
	50 µg/kg	16.0	7.3
	500 µg/kg	9.4	9.0
	1500 µg/kg	9.4	8.1
**Matrix effect (%)**		48	53

**Table 2:** The concentration of HGA and MCPrG in samples of different *Acer* species, peak areas of HGB and γ-glutamyl-MCPrG and HGB/HGA and γ-glutamyl-MCPrG/MCPrG peak area ratios. Data are presented as median (range).

Species	Sample Type	Concentration (mg/kg)		Peak Area		Peak Area Ratio	
		HGA	MCPrG	HGB	γ-glutamyl-MCPrG	HGB/HGA	γ-glutamyl-MCPrG/MCPrG
***A. pseudoplatanus* (sycamore maple)**	**Leaves**	512	11	1.3E+07	2.1E+07	0.31	9.9
	(n = 7)	(120–3202)	(6.3–158)	(1.0E+07–2.2E+08)	(3.4E+06–1.9E+08)	(0.08–1.1)	(0.21–22)
	**Seeds**	1333	146	1.2E+08	1.4E+08	0.90	5.4
	(n = 7)	(266–2962)	(35–267)	(8.6E+06–1.3E+09)	(1.1E+06–4.1E+08)	(0.03–29)	(0.08–46)
	**Seedlings**	3865	395	6.7E+08	3.0E+08	1.2	7.9
	(n = 2)	(3223–4508)	(290–500)	(5.8E+08–7.6E+08)	(2.4E+08–3.6E+08)	(1.2–1.3)	(7.4–8.5)
***A. negundo*** **(box elder maple)**	**Leaves**	535	3.4	1.7E+07	9.3E+06	0.26	26
	(n = 2)	(24–1047)	(2.7–4.1)	(1.0E+06–3.3E+07)	(3.4E+06–1.5E+07)	(0.24–0.29)	(22–29)
	**Seeds**	410	39	1.9E+08	1.7E+08	3.6	36
	(n = 2)	(236–584)	(22–56)	(1.6E+08–2.3E+08)	(7.6E+07–2.6E+08)	(2.9–4.3)	(30–42)
***A. platanoides* (Norway maple)**	**Leaves and seeds**	N.D.	N.D.	N.D.	N.D.	N.A.	N.A.
	(n = 3)						
***A. campestre*** **(field maple)**	**Leaves and seeds**	N.D.	N.D.	N.D.	N.D.	N.A.	N.A.
***A. tataricum*** **(Tatar maple)**	**Leaves and seeds**	N.D.	N.D.	N.D.	N.D.	N.A.	N.A.
***A. cappadocicum* (Cappadocian maple)**	**Leaves and seeds**	N.D.	N.D.	N.D.	N.D.	N.A.	N.A.

N.D. = not detected; N.A. = not analyzed.

## Data Availability

The data presented in this study are available in this article and Supplementary Materials.

## Supplementary Materials

The following supporting information can be downloaded at: https://www.mdpi.com/article/10.3390/toxins14090608/s1, Figure S1: The extraction of HGA in naturally contaminated *A. pseudoplatanus* seeds using 3 different solvents and 3 extraction cycles; Figure S2: Estimation of matrix effect. Calibration series of HGA (upper panel) and MCPrG (lower panel) standards in solvent, undiluted maple seed extract (matrix-matched standards, MMS) and 1 to 25 diluted maple seed extract; Figure S3: Overlaid MRM-extracted ion chromatograms of HGA (lower panel) and MCPrG (upper panel) in sycamore maple (*A. pseudoplatanus*) seedlings; Figure S4: HRMS-extracted ion chromatograms of MCPrG (upper panel) and γ-glutamyl-MCPrG (lower panel) in sycamore maple (*A. pseudoplatanus*) seeds; Figure S5: Structure elucidation using mzLogic algorithm. The query HRMS/MS spectrum was processed against structural and HRMS fragmentation databases. The ranked mzLogic results suggest hypoglycin B (HGB) as the top candidate; Figure S6: Structure elucidation using in silico fragmentation and fragment ion search (FISh) analysis of γ-glutamyl-MCPrG. Fragments in the query HRMS/MS spectrum were explained and structurally annotated using general fragmentation rules. The mass accuracy (∆ ppm) is shown for each fragment. The blue- and yellow-highlighted fragments are characteristic for MCPrG and glutamic acid, respectively; Table S1: Mass transitions and conditions for LC-MS/MS quantification of HGA and MCPrG in maple; Table S2: Method validation parameters for the determination of HGA and MCPrG in maple when samples were 1 to 25 diluted before LC-MS/MS measurement. LOD and LOQ were estimated using spiked blank material; Table S3: The concentration of HGA and MCPrG in leaves and seed samples of the same trees of sycamore and box elder maple, peak areas of HGB and γ-glutamyl-MCPrG and HGB/HGA and γ-glutamyl-MCPrG/MCPrG peak area ratios.
