# Supplementary material for: Co-Occurrence of Hypoglycin A and Hypoglycin B in Sycamore and Box Elder Maple Proved by LC-MS/MS and LC-HR-MS
Source: Toxins (Basel). 2022 Sep 1;14(9):608. doi: 10.3390/toxins14090608 (PMC9504185; doi:10.3390/toxins14090608)
Supplement: Supplementary file 1 [file toxins-14-00608-s001.zip › toxins-1839766-supplementary.pdf]

Supplementary Materials: Co-Occurrence of Hypoglycin A and Hypoglycin B in Sycamore and Box Elder Maple Proved by LC-MS/MS and LC-HR-MS

Ahmed H. El-Khatib, Anna Maria Engel and Stefan Weigel

Table S1. Mass transitions and conditions for LC-MS/MS quantification of HGA and MCPrG in maple.

Analyte	Precursor ion (*m*/*z*)	DP (V)	EP (V)	Product ions (*m*/*z*)	CE (V)	CXP (V)	Dwell Time (ms)	Expected RT (min)
**MCPrG**	128.0	35.8	4.45	64.8 (quant.)	25.76	7.0	80	1.82
				92.0	20.50	14.0		
				66.9	32.67	7.0		
**HGA**	142.0	15.0	2.6	73.9 (quant.)	11.00	8.0		3.18
				95.9	11.00	4.8		
				46.2	18.50	9.0		

DP: Declustering Potential, EP: Entrance Potential, CE: Collision Energy, CXP: Collision Cell Exit Potential, RT: Retention Time.

Table S2. Method validation parameters for the determination of HGA and MCPrG in maple when samples were 1to25 diluted before LC-MS/MS measurement. LOD and LOQ are estimated using spiked blank material.

Parameter		HGA	MCPrG
**Calibration range (ng/mL)**		0.5-100 (250-50000 μg/kg)	0.5-100 (250-50000 μg/kg)
**Correlation coefficient (r)**		0.9995	0.9996
**LOD (μg/kg)**		28.2	154
**LOQ (μg/kg)**		93.0	507
**Recovery (%)**	500 μg/kg	92.1	109
	1500 μg/kg	90.4	102
**Repeatability (RSD_r) (%)**	500 μg/kg	4.9	13.3
	1500 μg/kg	10.0	4.9
**Within-laboratory reproducibility (RSD_{wR}) (%)**	500 μg/kg	8.9	19.4
	1500 μg/kg	8.0	7.8
**Matrix effect (%)**		99	109

Table S3. The concentration of HGA and MCPrG in leaves and seed samples of the same trees of sycamore and box elder maple, peak areas of HGB and γ-glutamyl-MCPrG and HGB/HGA and γ-glutamyl-MCPrG/MCPrG peak area ratios.

Species	Sample #	Sample type	Concentration (mg/kg)		Peak area		Peak area ratio	
			HGA	MCPrG	HGB	γ-glutamyl-MCPrG	HGB/HGA	γ-glutamyl-MCPrG/MCPrG
A. pseudoplatanus (Sycamore maple)	1	Leaves	1011	11	1.0E+07	1.7E+07	0.08	9.9
		Seeds	1333	85	3.4E+08	4.1E+08	2.0	46
	2	Leaves	642	124	2.6E+07	2.4E+07	0.31	1.9
		Seeds	2327	217	1.2E+08	1.4E+08	0.41	6.3
	3	Leaves	120	9.7	1.0E+07	2.1E+07	0.63	12
		Seeds	2962	265	3.5E+08	1.4E+08	0.90	5.4
	4	Leaves	459	6.3	1.3E+07	3.6E+06	0.18	3.3
		Seeds	303	35	6.2E+07	2.3E+07	1.3	6.0
A. negundo (Box elder maple)	1	Leaves	1047	4.1	3.3E+07	1.5E+07	0.24	22
		Seeds	584	56	2.3E+08	2.6E+08	2.9	42
	2	Leaves	24	2.7	1.0E+06	3.4E+06	0.29	29
		Seeds	236	22	1.6E+08	7.6E+07	4.3	30

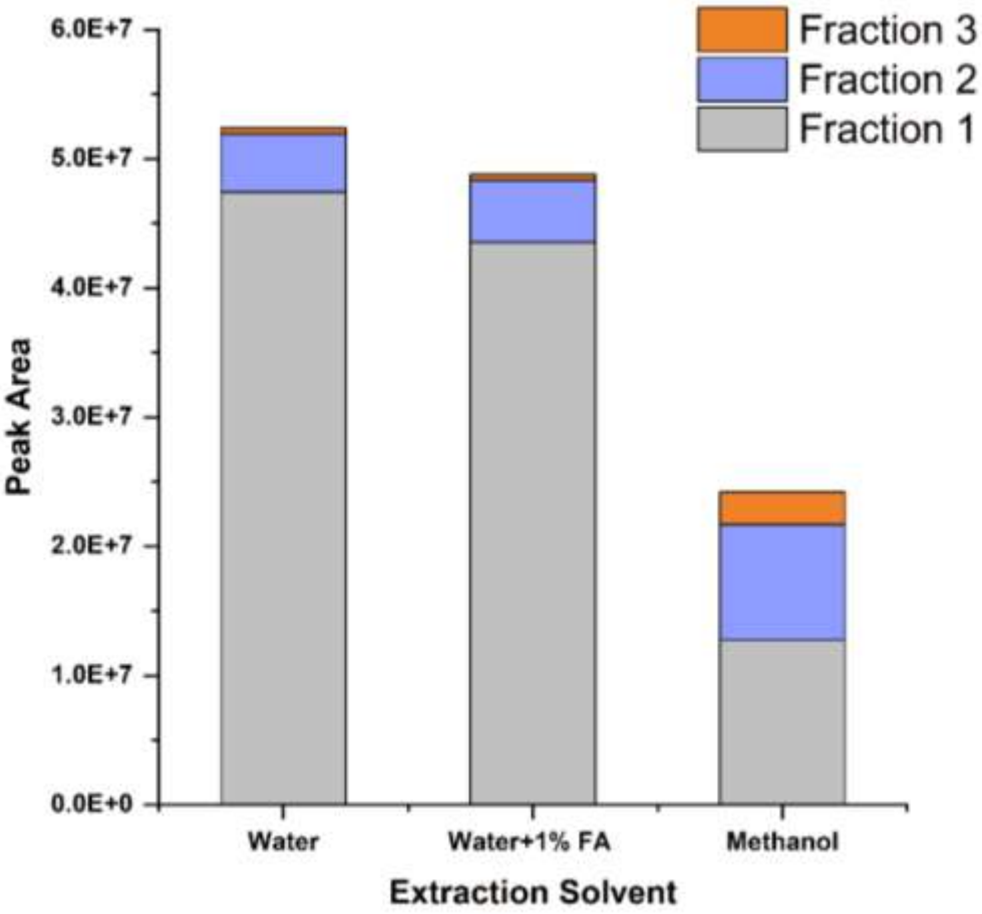

Figure S1. The extraction of HGA in naturally contaminated *A. pseudoplatanus* seeds using 3 different solvents and 3 extraction cycles.

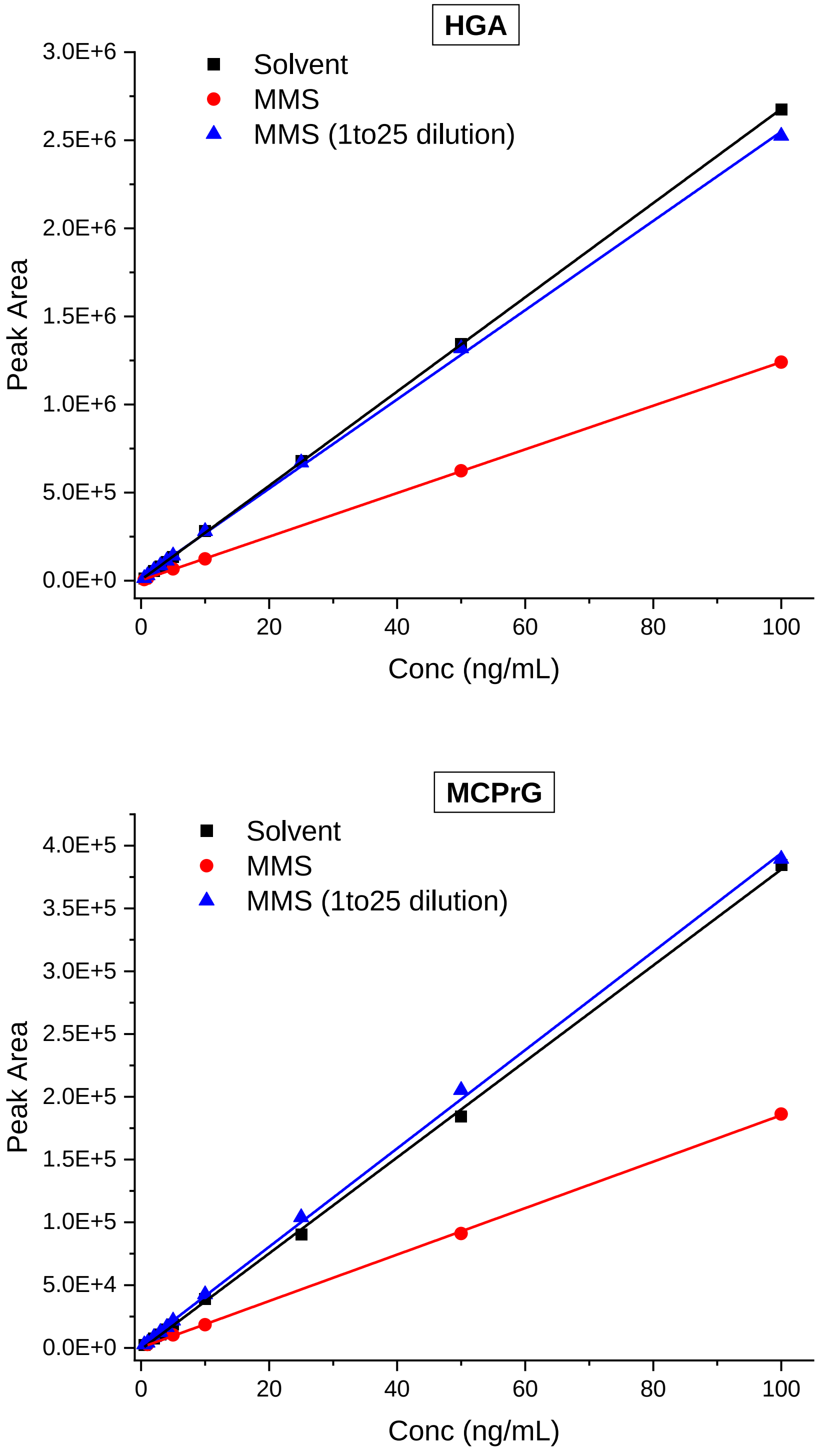

Figure S2. Estimation of matrix effect. Calibration series of HGA (upper panel) and MCPrG (lower panel) standards in solvent, undiluted maple seed extract (matrix-matched standards, MMS) and 1to25 diluted maple seed extract.

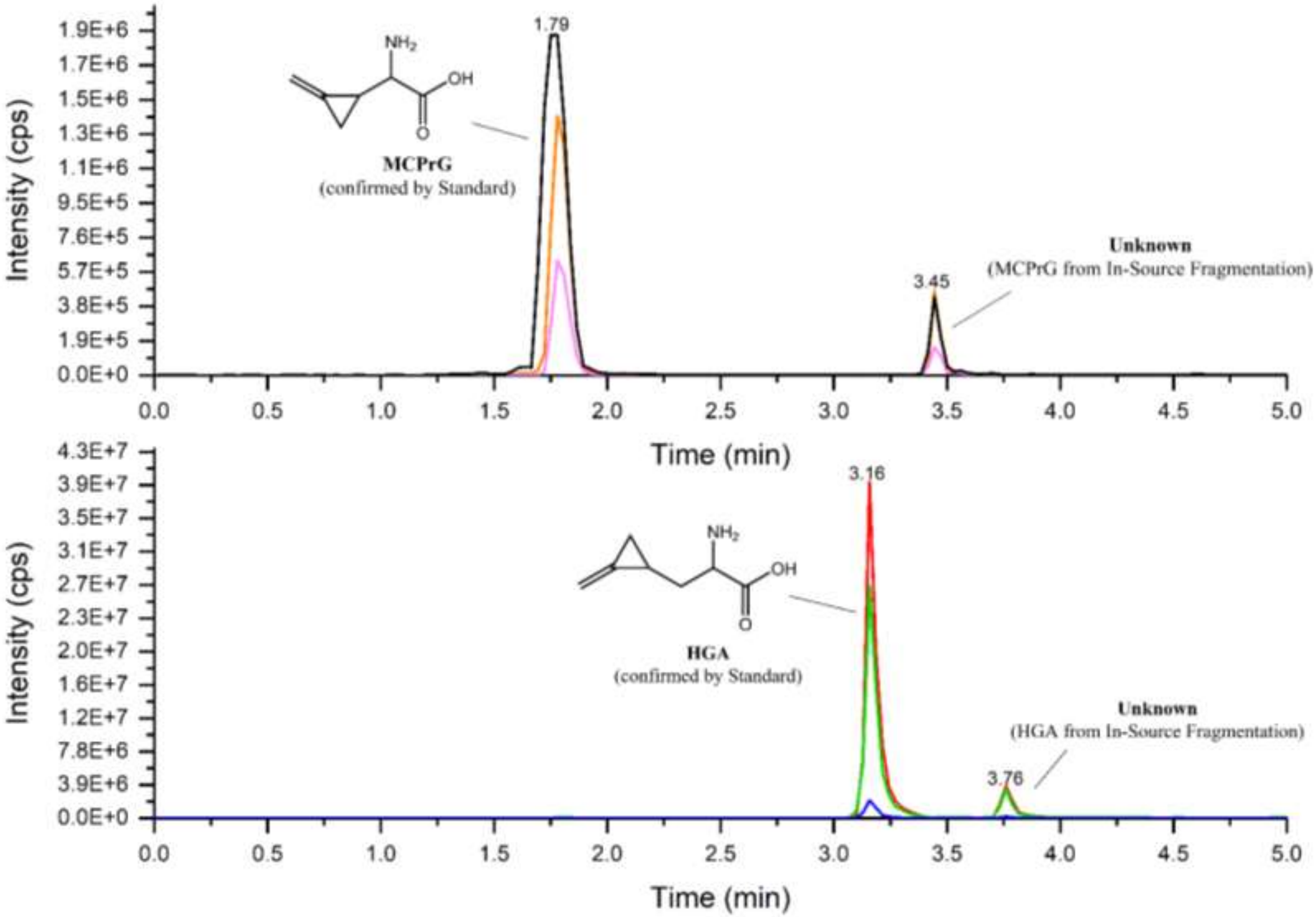

Figure S3. Overlaid MRM extracted ion chromatograms of HGA (lower panel) and MCPrG (upper panel) in sycamore maple (*A. pseudoplatanus*) seedlings.

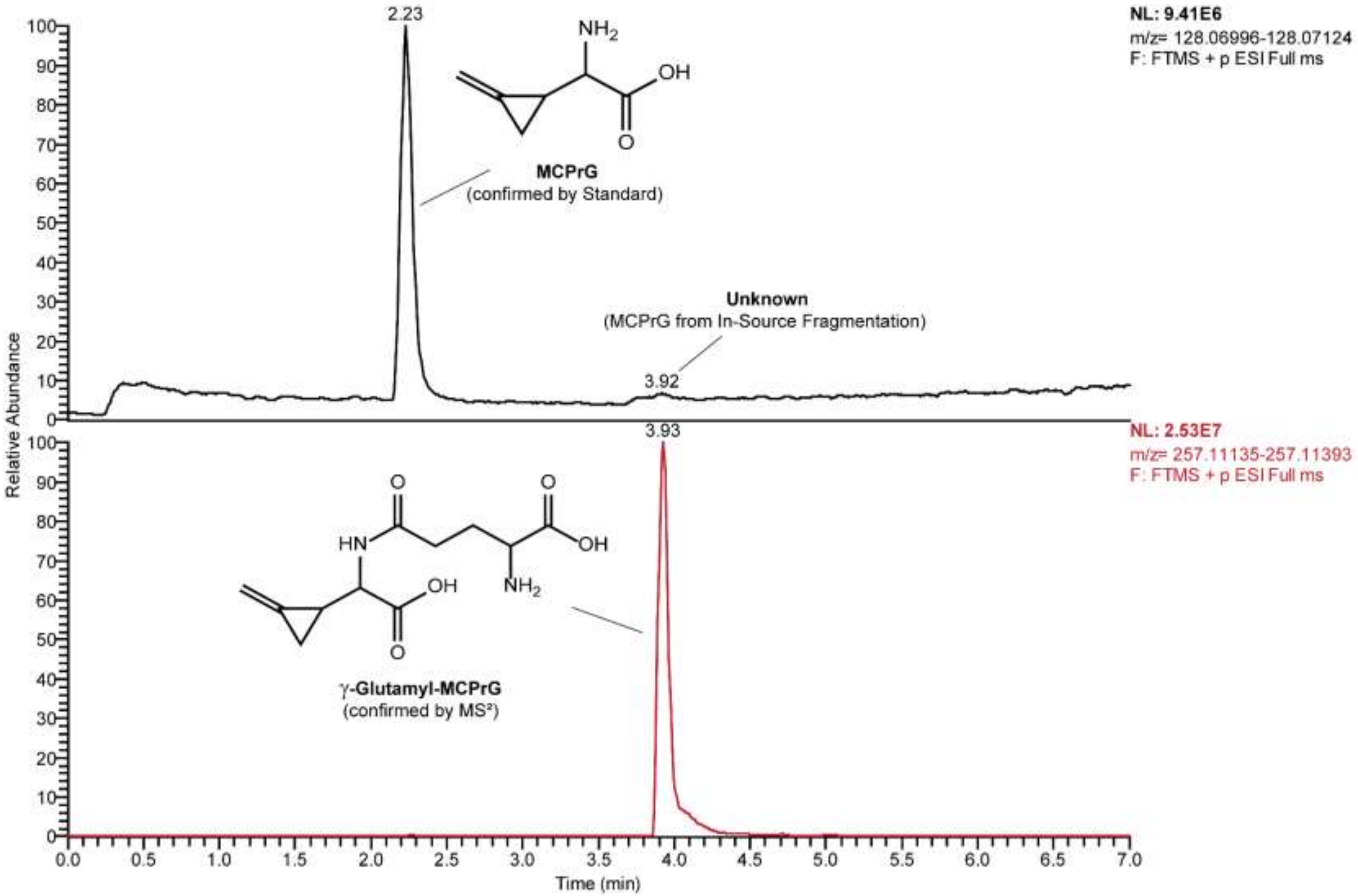

Figure S4. HRMS extracted ion chromatograms of MCPrG (upper panel) and γ-glutamyl-MCPrG (lower panel) in sycamore maple (*A. pseudoplatanus*) seeds.

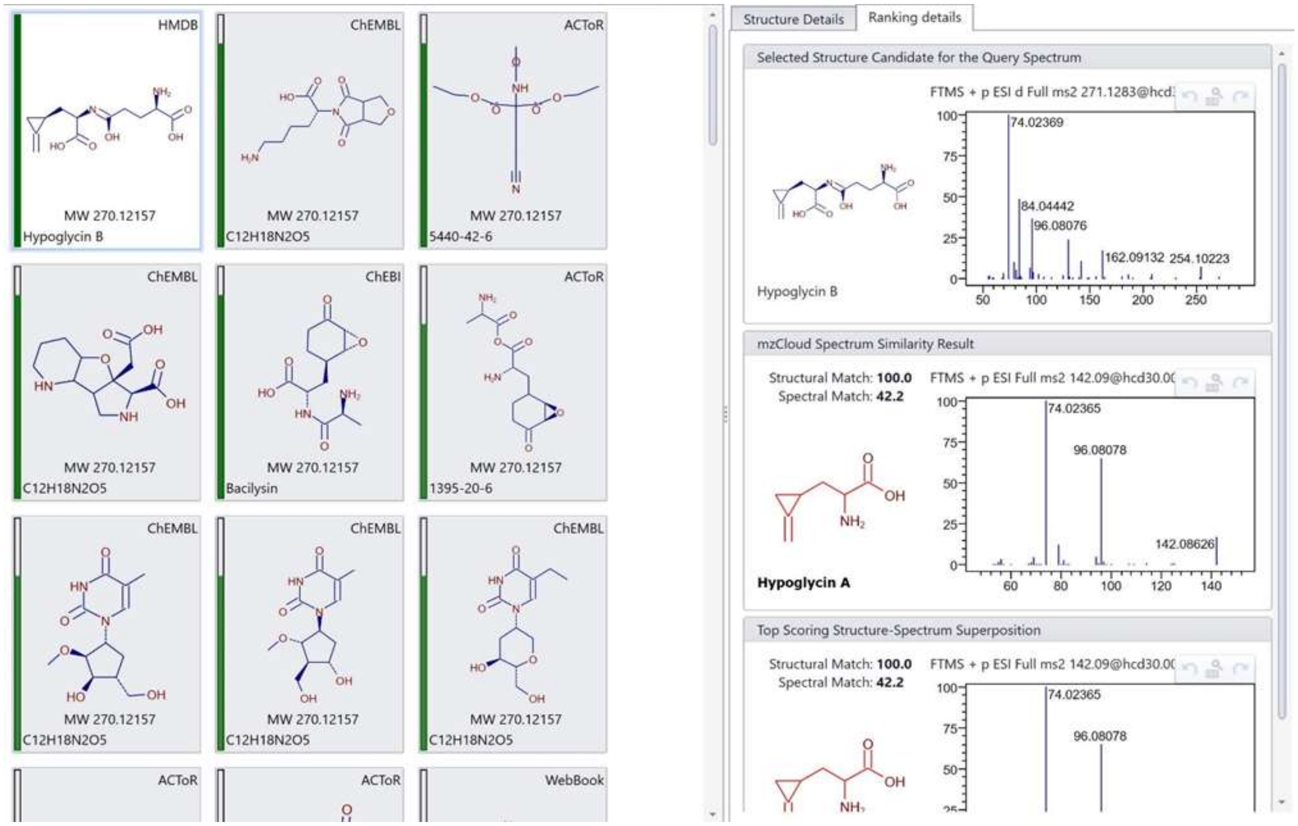

Figure S5. Structure elucidation using mzLogic algorithm. The query HRMS/MS spectrum was processed against structural and HRMS fragmentation databases. The ranked mzLogic results suggest hypoglycin B (HGB) as the top candidate.

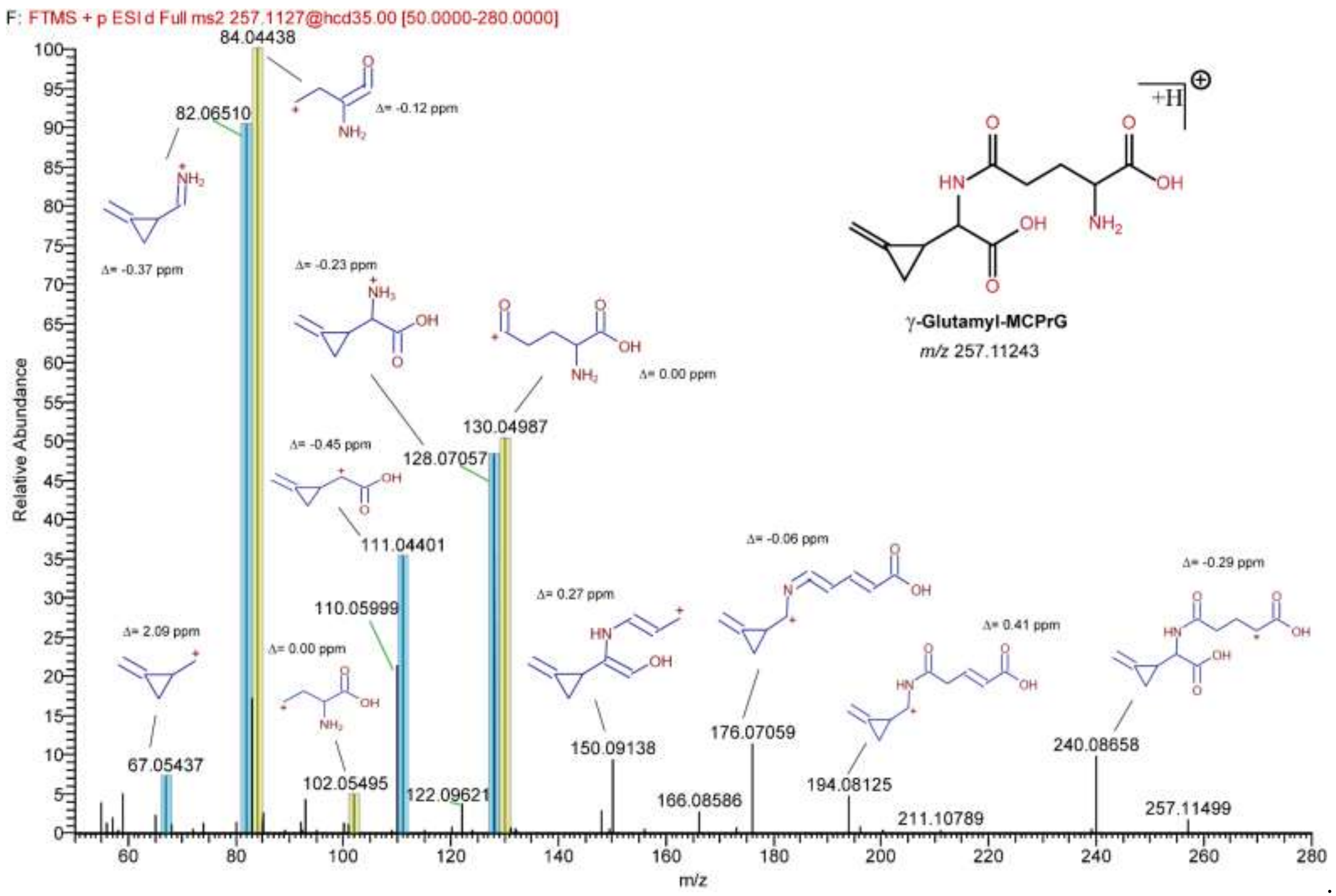

Figure S6. Structure elucidation using *in silico* fragmentation and Fragment Ion Search (FISh) analysis of γ-glutamyl-MCPrG. Fragments in the query HRMS/MS spectrum were explained and structurally annotated using general fragmentation rules. The mass accuracy (Δ ppm) is shown for each fragment. The blue- and yellow-highlighted fragments are characteristic for MCPrG and glutamic acid, respectively.
